# Circadian Regulation of Myocardial Sarcomeric *Titin-cap* (*Tcap*, *Telethonin*): Identification of Cardiac Clock-Controlled Genes Using Open Access Bioinformatics Data

**DOI:** 10.1371/journal.pone.0104907

**Published:** 2014-08-14

**Authors:** Peter S. Podobed, Faisal J. Alibhai, Chi-Wing Chow, Tami A. Martino

**Affiliations:** 1 Cardiovascular Research Group, Department of Biomedical Sciences, University of Guelph, Guelph, Ontario, Canada; 2 Department of Molecular Pharmacology, Albert Einstein College of Medicine, Bronx, New York, United States of America; University of Texas Southwestern Medical Center, United States of America

## Abstract

Circadian rhythms are important for healthy cardiovascular physiology and are regulated at the molecular level by a circadian clock mechanism. We and others previously demonstrated that 9–13% of the cardiac transcriptome is rhythmic over 24 h daily cycles; the heart is genetically a different organ day versus night. However, which rhythmic mRNAs are regulated by the circadian mechanism is not known. Here, we used open access bioinformatics databases to identify 94 transcripts with expression profiles characteristic of CLOCK and BMAL1 targeted genes, using the CircaDB website and JTK_Cycle. Moreover, 22 were highly expressed in the heart as determined by the BioGPS website. Furthermore, 5 heart-enriched genes had human/mouse conserved CLOCK:BMAL1 promoter binding sites (E-boxes), as determined by UCSC table browser, circadian mammalian promoter/enhancer database PEDB, and the European Bioinformatics Institute alignment tool (EMBOSS). Lastly, we validated findings by demonstrating that *Titin cap* (*Tcap*, *telethonin*) was targeted by transcriptional activators CLOCK and BMAL1 by showing 1) *Tcap* mRNA and TCAP protein had a diurnal rhythm in murine heart; 2) cardiac *Tcap* mRNA was rhythmic in animals kept in constant darkness; 3) *Tcap* and control *Per2* mRNA expression and cyclic amplitude were blunted in Clock^Δ19/Δ19^ hearts; 4) BMAL1 bound to the *Tcap* promoter by ChIP assay; 5) BMAL1 bound to *Tcap* promoter E-boxes by biotinylated oligonucleotide assay; and 6) CLOCK and BMAL1 induced *tcap* expression by luciferase reporter assay. Thus this study identifies circadian regulated genes *in silico*, with validation of *Tcap,* a critical regulator of cardiac Z-disc sarcomeric structure and function.

## Introduction

The circadian clock mechanism is an important regulator of cardiovascular physiological and biochemical processes (reviewed in [1]–[4]). The molecular circadian mechanism, at its most basic level, is a 24 h transcription and translation feedback loop (reviewed in [5]–[7]). The positive arm consists of a heterodimeric pairing of two key basic-helix-loop-helix domain proteins termed circadian locomotor output cycles kaput (CLOCK), and muscle arnt like protein 1 (BMAL1). CLOCK and BMAL1 heterodimers bind to promoter E-box elements to induce expression of their repressors called PERIOD (PER) and CRYPTOCHROME (CRY). The molecular mechanism is cell autonomous, and cardiac expression of these core mechanism genes was first demonstrated in rat [8] and human [9] hearts by polymerase chain reaction (PCR) [8], and rat heart explants by luciferase assay [10].

The circadian mechanism may also regulate a wide variety of additional genes, as a total of 462 out of 5,120 cardiac genes analyzed (∼9%) were rhythmically expressed in murine heart under endogenous circadian conditions, by Affymetrix oligonucleotide microarray analyses [11]. Moreover, since mammals including humans live in a diurnal (24 h day/night) and not a circadian environment, we demonstrated that 1,634 out of 12,488 genes (∼13%) in murine heart were rhythmic under regular 24 h diurnal conditions, by microarray and bioinformatics analyses [12]–[14]. However, rhythmic gene expression does not necessarily constitute direct regulation by the molecular circadian mechanism. Moreover, the composition of rhythmic genes (other than core clock mechanism genes) is tissue-specific, underlying the structure and function of that tissue. Comparison of microarray data from murine heart versus liver revealed that only 52 rhythmic genes were common to both organs [11], thus supporting this notion. To date, which of the 9–13% of rhythmic heart genes are targets of the circadian transcriptional activators CLOCK and BMAL1 is not known.

In this study, we developed a novel *in silico* analysis workflow approach using open access bioinformatics databases to identify putative CLOCK and BMAL1 transcriptionally regulated cardiac genes. We validated our approach by demonstrating that rhythmic expression of the cardiac sarcomeric *Titin cap* gene (*Tcap*, *telethonin*, a critical regulator of cardiac Z-disc sarcomeric structure and function [15]–[17]) is under circadian control. This bioinformatics driven investigation can be applied to identify tissue-specific circadian regulated genes.

## Methods

### Animals

All animal work was conducted under the guidelines of the Canadian Council on Animal Care, and approved by the Animal Use Protocols at the University of Guelph. C57Bl/6N mice (nomenclature C57BL/6NCrl, Charles River Laboratories, Quebec, Canada) were housed in a normal diurnal 12 h light (L) and 12 h dark (D) environment (with food and water available *ad libitum,* light intensity maintained at 100–200 lux (unless otherwise noted), room temperature of 22°C–24°C). To investigate diurnal *Tcap* mRNA and TCAP protein rhythms, 8 week old male C57Bl/6N mice were euthanized with CO_2_ and cervical dislocation every 4 h across the diurnal cycle starting at 1 h before lights ON (Zeitgeber Time, ZT = 23, n = 6/time point). Hearts were immediately frozen in liquid nitrogen and stored at −80°C until use. To investigate whether a mutation in the circadian mechanism played a role in *Tcap* rhythms, CLOCK-mutant mice were used. The heterozygote Clock^+/Δ19^ (isogenic C57BL/6J background) founder mice [18], kindly supplied by Dr. Erik Herzog (Washington University) and Dr. Joseph S. Takahashi (University of Texas Southwestern), were used to produce Clock^Δ19/Δ19^ progeny (homozygous for the CLOCK point mutation). Eight week old homozygous male Clock^Δ19/Δ19^ and wild type (WT) littermates were housed in the normal diurnal 12∶12 L:D environment, then for the experiment they were transferred into constant darkness (D:D, dim red light at <1 lux under a Bludgeon Red filter (AP8350, Apollo design technology, Fort Wayne, USA)). Starting at 30 h after transfer into D:D (CT = 18), mice were euthanized as described above, and hearts collected every 4 h from WT littermate (CT18 to CT62) and Clock^Δ19/Δ19^ (CT22 to CT46) mutants (n = 4/time point). To investigate BMAL1 binding to E-boxes in the *Tcap* promoter, an additional set of C57Bl/6J mice were transferred into constant darkness (D:D, dim red light at <1 lux) for 38 h (Circadian Time, CT = 26) or 50 h (CT = 38), then euthanized and hearts collected for chromatin immunoprecipitation (ChIP) assay (n = 3/time point).

### CircaDB gene expression website and JTK_Cycle analysis

The Circadian Expression Profiles Data Base (CircaDB, http://bioinf.itmat.upenn.edu/circa) is an open access bioinformatics website that illustrates rhythmic gene expression from microarrays [19]–[22]. The embedded JTK_Cycle algorithm plots expression level, period, phase, amplitude, JTK p-value (estimates the probability for rejecting the null hypothesis that the target gene expression was not circadian), and JTK q-value (estimates the false discovery rate for considering the gene circadian). We interrogated the Mouse 1.OST Heart (Affymetrix) microarrays, using the probability filter JTK P-value, a cut-off value of 0.001, and JTK phase range of 0–40. Genes were selected for further interrogation if they had a period of 23 h to 25 h, and a phase of 16 h to 20 h. This profile was chosen as it was consistent with the profiles of known CLOCK and BMAL1 transcription targets (*Per1* [23], *Per2* [24], *Per3* [25], *Rev-Erbβ* [26] and *Dbp* [27]), which were used as reference guides.

### BioGPS gene annotation and expression analyses

To determine whether the genes identified as rhythmic by CircaDB exhibited high levels of expression in the heart, the BioGPS website (http://biogps.org/) was used [28]. The embedded GeneAtlas MOE430 gcrma gene expression activity chart [29] was used to interrogate gene expression data from up to 96 murine cell types and tissues, and the BioGPS Plugin Library allowed for further investigation of genes of interest including Gene Ontology (GO) analyses.

### *In silico* Circadian Motif search

The University of California Santa Cruz (UCSC) table browser tool (http://genome.ucsc.edu/cgi-bin/hgTables) [30] was used to search the 1000 base pair (bp) region upstream of the transcriptional start site (TSS) for genes of interest (http://genome.ucsc.edu/cgi-bin/hgTables). Promoter regions were searched for putative circadian E-box elements that could be used for CLOCK and BMAL1 transcriptional regulation, as described previously [14], [31]. Identified sequences were further interrogated to determine if they were conserved in mouse vs. human genes, by the circadian mammalian promoter/enhancer database (PEDB, http://promoter.cdb.riken.jp/circadian/html) [32], complemented with the European Bioinformatics Institute pairwise alignment tool (EMBOSS, http://www.ebi.ac.uk/Tools/psa/emboss_water/nucleotide.html) [33].

### Quantitative RealTime Polymerase Chain Reaction (qRTPCR)

Total RNA was isolated from hearts using TRIZOL (Invitrogen) as previously described [14], [31] and quality assessed by Nanodrop ND1000 (Thermo-Scientific). Amplification was performed on a VIIA7 Real time PCR system (Applied Biosystems) using the Power SYBR Green RNA to CT 1-Step PCR kit (Applied Biosystems) according to the manufacturer’s specifications. Primers for *Tcap* (forward, 5′-CCTTCTGGGCTGAGTGGAAA-3′; reverse, 5′-CTGCCGGTGGTAGGTCTCAT-3′), *Per2* (forward, 5′-TCATCATTGGGAGGCACAAA-3′; reverse, 5′-GCATCAGTAGCCGGTGGATT-3′), and *histone* (forward, 5′-GCAAGAGTGCGCCCTCTACTG-3′; reverse, 5′-GGCCTCACTTGCCTCCTGCAA-3′) were designed with Primer Express (Applied Biosystems). PCR samples were run in triplicate and values were normalized to *histone* using the delta delta CT method.

### Protein Purification

Soluble heart proteins were collected following tissue homogenization using a Potter-Elvehjem tissue grinder and ice-cold Urea/CHAPs lysis buffer (10 mM Tris pH 8, 8 M Urea, 4% w/v 3-[(3-cholamidopropyl) dimethylammonio]-1-propanesulfonate), with protease inhibitors (Roche, complete Mini EDTA-free). Homogenates were pelleted (4°C, 10 min, 12,000×g), supernatant collected, and protein concentration measured by Bradford assay (BioRad).

### SDS-PAGE and Western Blot

Protein extracts (10 µg) were separated on 12.5% sodium dodecyl sulfate polyacrylamide gel electrophoresis (SDS-PAGE) and transferred to a PVDF membrane (BioRad). The membranes were blocked for 1 h at room temperature with 5% dried non-fat milk in 5% TBST (10 mM Tris [pH 8.0], 150 mM NaCl, and 0.05% Tween-20), then washed 2x in 5% TBST, and incubated overnight at 4°C with monoclonal mouse anti-TCAP antibody (1∶2000; BD, Cat #612328). Immunoreactive bands were visualized with horseradish peroxidase-conjugated goat anti-mouse IgG (1∶5000; Sigma, A2304) and Clarity reagent (BioRad) on a Chemidoc MP system (BioRad). Anti-β-ACTIN antibody (1∶25000; Millipore, MAB1501) was used as a loading control.

### ChIP assay

Hearts were collected at the appropriate circadian times (CT26, *Tcap* mRNA expression increasing, or CT38, *Tcap* mRNA decreasing). ChIP was performed using a Magna ChIP G kit (Millipore, 17–611) according to the manufacturer’s specifications. Briefly, chromatin was sonicated using Sonic Dismembrator Model 100 (Fisher) to yield ∼500 bp fragments that were confirmed by ethidium bromide agarose gel electrophoresis. DNA was quantified using the Nanodrop ND1000 (Thermo-Scientific) and 50 µg was used for immunoprecipitation. For input, 1% of the chromatin was saved prior to immunoprecipitation. Chromatin was pre-cleared for 1 h at 4°C using 20 µl of magnetic beads, then incubated with rabbit polyclonal anti-BMAL1 (Abcam, ab3350), or control rabbit IgG (Millipore, 12–370) antibody overnight at 4°C. DNA was purified using the kit spin columns, and quantified by PCR using the Fast SYBR Green Master Mix (Quanta Bioscience) and VIIA7 PCR system (Applied Biosystems) under the following conditions; 95°C for 10 min, followed by 40 cycles of 95°C for 5 sec and 60°C for 20 sec, using the *Tcap* ChIP primers (forward 5′-CCCATCACCACCAGTGAGTCT-3′; reverse 5′-GCCCTTTAAATAGCCCCTTCTTC-3′). DNA abundance was quantified as percent input.

### DNA precipitation assays

DNA precipitation assays were performed as described previously [34]. Briefly, COS cells were transfected with the expression vectors for BMAL1 and harvested in Triton-lysis buffer (20 mM Tris pH 7.4, 134 mM NaCl, 2 mM EDTA, 25 mM β-glycerophosphate, 2 mM NaPPi, 10% glycerol, 1% Triton X-100, 1 mM phenylmethylsulfonyl fluoride, 1 mM benzamidine and 10 µg/ml leupeptin). Double-stranded biotinylated oligonucleotides for the *Tcap* promoter [–749 bp E box, 5′-Biotin-AAAATAGAGCCAGCTTGAGGCTGGTCTGGATCTCC-3′; −274 bp E box, 5′-Biotin- GCCAAGGTGGCACGAGGCTGCCCATGTGCCTGG TCCGAG −3′; −74 bp E box, 5′-Biotin- CTGCTTATAGCATCTGACGCCAGAGGGGCTGAAAATAG −3′] were incubated with BMAL1 and CLOCK cell extracts for 12 h before precipitation with 20 µl of streptavidin-Sepharose for 2 h. After three washes in Triton-lysis buffer, precipitated DNA and associated proteins were separated by SDS-PAGE, and an immunoblot was performed to detect BMAL1.

### Luciferase assays

The *Tcap* promoter was amplified from mouse genomic DNA and subcloned into the pGL3 basic luciferase reporter plasmid using MluI and XhoI sites. Expression vectors for *Bmal1* and *Clock* (50 ng) were co-transfected with the *Tcap* luciferase reporter plasmids (100 ng) and control plasmid pRSV β-galactosidase (25 ng) into COS cells. Transfected cells were serum shocked (20%) for 2 h and maintained in 2% media for 24 h before harvest. Data were presented as relative luciferase activity, calculated as the ratio of the luciferase activity to the activity of β-galactosidase.

### Statistical Analysis

Values are expressed as mean ± SEM. Statistical comparisons of gene expression (ChIP assay, luciferase activity) were performed using SPSS software v.21 (IBM) and a two-tailed Students t-test, and diurnal rhythms in mRNA or protein were analyzed using the JTK_Cycle nonparametric algorithm as described on the CircaDB database (http://bioinf.itmat.upenn.edu/circa) [19]–[21]. For JTK_Cycle the p-value, phase, and amplitude were evaluated, but not period as this requires at least 3 cycles of data [35].

## Results

**Figure 1:**
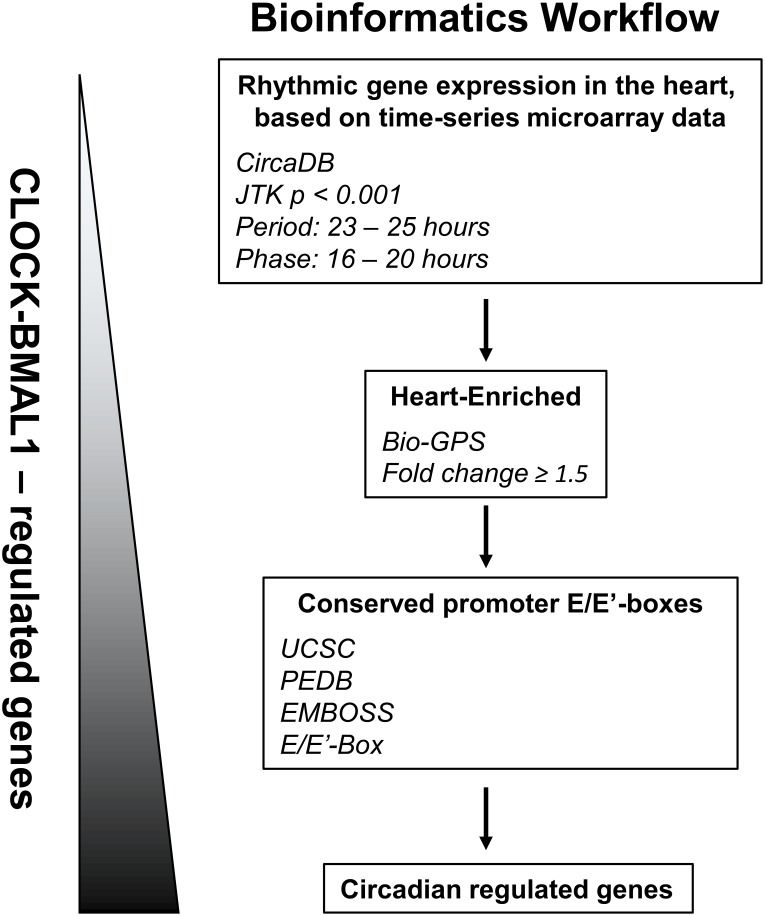
Novel bioinformatics workflow to identify circadian regulated cardiac genes. Distinct patterns of circadian gene expression in the heart were visualized using the Circadian Expression Profiles Data Base (CircaDB) and embedded JTK_Cycle algorithm [19]–[22] using the JTK_Cycle parameters p<0.001, period 23 h to 25 h, and phase 16 h to 20 h. The gene list was further enriched for high cardiac mRNA expression levels, using the BioGPS website and GeneAtlas MOE430 gene expression/activity display [28], [29]. Genes were selected if they exhibited a ≥1.5 fold increased expression in heart. An *in silico* circadian motif search was then performed to detect conserved E-box elements in the gene promoter regions that could be used for CLOCK and BMAL1 transcription, using the University of California Santa Cruz (UCSC) table browser tool [30], the circadian mammalian promoter/enhancer database (PEDB) [32], and the European Bioinformatics Institute pairwise nucleotide alignment tool (EMBOSS) [33]. Circadian regulation of candidate gene *Tcap* was investigated experimentally using *in vivo* and *in vitro* approaches.

To identify rhythmic cardiac genes that might be under circadian transcriptional regulation, we designed a workflow based on open access bioinformatics databases (Figure 1). First, rhythmic expression profiles of murine heart genes were obtained from the online CircaDB database and the JTK_Cycle algorithm [19]–[22]. The reference genes we used as a guide were *Per1* [23], *Per2* [24], *Per3* [25], *Nr1d2* (*Rev-Erbβ*) [26] and *Dbp* [27]), (see Table 1). Based on these data, we then searched CircaDB for additional genes that might be transcriptionally regulated in a similar manner, using the search parameters of p<0.001, period = 23 h to 25 h, and phase = 16 h to 20 h. This produced a list of 94 genes with robust circadian expression profiles (Table S1).

**Table 1:** Reference Genes.

Guide Genes	JTK p-Value	JTK q-Value	Period	Phase
***Per1***	0.00179	0.0575019	24	16
***Per2***	2.48E-10	1.47E-06	24	19
***Per3***	1.26E-11	1.49E-07	24	18
***Nr1d2***	3.28E-09	6.85E-06	24	17
***Dbp***	5.34E-08	4.63E-05	24	16.5

Reference genes were identified on CircaDB and the JTK_Cycle algorithm, and Mouse 1.OST Heart (Affymetrix) microarrays [19]–[22]. The reference genes used as a guide were *Per1* [23], *Per2* [24], *Per3* [25], *Nr1d2 (Rev-Erbβ)* [26] and *Dbp* [27]), as CLOCK and BMAL heterodimers are involved in their transcription.

Next, we investigated which of the 94 putative circadian regulated genes were highly expressed in heart tissue. To do this, each of the genes was searched on the BioGPS website, using the GeneAtlas MOE430 gene expression/activity module. As shown in Table 2, there were 22 transcripts (23%) that exhibited ≥1.5 fold increased expression in the heart, as compared to the mean expression values of all murine tissues in the datasets. The gene symbols, cardiac enrichment (fold change), and JTK_Cycle values for these 22 genes are provided in Table 2, while additional details on the microarray probe ID, and cardiac versus average murine expression levels are in Table S2.

**Table 2:** The 22 Rhythmic Genes Highly Expressed in Heart vs. 96 Murine Tissues.

Gene Symbol	Heart-enriched (fold change)	JTK p-value	JTK q-value	JTK Period	JTK Phase
***Rhobtb1***	12.8	1.229E-10	8.741E-07	24	19
***Tcap***	10.1	1.053E-08	1.628E-05	24	16
***Mlf1***	9.2	5.973E-07	2.124E-04	24	17.5
***Mylk4***	7.5	2.364E-07	1.274E-04	24	18
***Ccdc141***	6.3	3.222E-04	1.776E-02	24	16
***Kcnh2***	6.1	2.364E-07	1.274E-04	24	18
***Sh3rf2***	5.2	5.841E-04	2.683E-02	24	17
***Gpcpd1***	4.5	3.781E-07	1.660E-04	23	18
***Calcoco1***	4.2	7.792E-04	3.326E-02	24	18
***Ppip5k2***	4.0	1.257E-04	9.066E-03	24	19
***Vps25***	3.9	3.244E-05	3.516E-03	24	18
***Raph1***	3.9	5.841E-04	2.683E-02	24	20
***Nfia***	3.6	3.244E-05	3.516E-03	24	16
***Socs2***	3.5	1.732E-04	1.153E-02	24	18
***Cygb***	3.3	1.080E-05	1.685E-03	24	17
***Klf9***	3.2	6.482E-05	5.748E-03	24	16
***Usp2***	2.6	9.323E-07	2.934E-04	24	18
***Dusp7***	2.4	1.732E-04	1.153E-02	25	19
***1810013L24Rik***	2.3	3.222E-04	1.776E-02	23	18
***Acer2***	1.9	4.603E-05	4.547E-03	24	18
***Slco5a1***	1.8	3.781E-07	1.660E-04	24	20
***Timp3***	1.6	9.430E-10	3.353E-06	23	20

Heart-enriched (fold change) values are derived from the BioGPS website (http://biogps.org/) [28] and embedded GeneAtlas MOE430 gcrma gene expression activity chart [29], which were used to interrogate cardiac expression of genes of interest. Statistical values are from CircaDB and the JTK_Cycle algorithm, Mouse 1.OST Heart (Affymetrix) microarrays [19]–[22].

Furthermore, to determine whether these 22 cardiac enriched rhythmic gene transcripts might be under circadian mechanism control, we searched promoter sequences (retrieved from UCSC; http://genome.ucsc.edu/cgi-bin/hgTables) within 1000****bp of the TSS for CLOCK and BMAL1 E-box binding elements. Moreover we investigated whether these sequences were phylogenetically conserved between mouse and human, based on the circadian mammalian promoter enhancer database (PEDB, http://promoter.cdb.riken.jp/circadian/html) [32] along with the European Bioinformatics Institute alignment tool (EMBOSS; www.ebi.ac.uk/Tools/psa/emboss_water/nucleotide.html) [33]. The rationale for comparing different species was to enrich for candidate genes with evolutionarily conserved biological functions [32], [36]. We also selected for genes that had tandem E1–E2 box elements as these and proper spacing are implicated in CLOCK and BMAL1 mediated transcription [37]–[39]. As shown in Table 3, conserved tandem motifs were noted for *Tcap*, *Rhobtb1*, *Ccdc141*, *Kcnh2*, and *Dusp7*.

**Table 3:** Heart-enriched rhythmic proteins with human-mouse conserved E-box motifs.

Gene Symbol	Mouse		Human		Mouse Sequence: Human Sequence
	from TSS	Refseq ID	from TSS	Refseq ID	
*Tcap*	−74	NM_011540	−77	NM_003673	tagcatctgacgccagaggggc |||||||||||.|||||||||| tagcatctgacaccagaggggc
*Tcap*	−274	NM_011540	−246	NM_003673	cacgaggctgcccatgtgcct |||.||||||..||||||.|| cacaaggctgggcatgtggct
*Rhobtb1*	−12	NM_001081347 (V1)	−464	NM_014836 (V1)	cagcgcttagtcagcagctggg ||||.||||||||||||||||| cagcccttagtcagcagctggg
*Rhobtb1*	−12	NM_001081347 (V1)	−464	NM_001242359 (V4)	cagcgcttagtcagcagctggg ||||.||||||||||||||||| cagcccttagtcagcagctggg
*Rhobtb1*	−62	NM_001081347 (V1)	−517	NM_014836 (V1)	agcacgtgcagccgcccagggag ||||||||||||||||||||||| agcacgtgcagccgcccagggag
*Rhobtb1*	−62	NM_001081347 (V1)	−517	NM_001242359 (V4)	agcacgtgcagccgcccagggag ||||||||||||||||||||||| agcacgtgcagccgcccagggag
*Rhobtb1*	−549	NM_001252638 (V4)	−464	NM_014836 (V4)	cagcgcttagtcagcagctggg ||||.||||||||||||||||| cagcccttagtcagcagctggg
*Rhobtb1*	−549	NM_001252638(V4)	−464	NM_001242359 (V4)	cagcgcttagtcagcagctggg ||||.||||||||||||||||| cagcccttagtcagcagctggg
*Ccdc141*	−872	NM_001025576	−896	NM_173648	tgacaggg-ctccaacagctgct ||||||||.||||||||||||| tgacagggaatccaacagctgct
*Kcnh2*	−713	NM_013569	−733	NM_000238 (V1)	tacagctaaaaggccgcaactgc |.||||||..||||||||||||| tgcagctaggaggccgcaactgc
*Kcnh2*	−713	NM_013569	−733	NM_172056 (V2)	tacagctaaaaggccgcaactgc |.||||||..||||||||||||| tgcagctaggaggccgcaactgc
*Kcnh2*	−320	NM_013569	−250	NM_000238 (V1)	caaaccgaccatcgcacctgtc |||.|||.|||||||||||||| caagccggccatggcacctgtc
*Kcnh2*	−320	NM_013569	−250	NM_172056 (V2)	caaaccgaccatcgcacctgtc |||.|||.||||.||||||||| caagccggccatggcacctgtc
*Dusp7*	−691	NM_153459	−877	NM_001947	gtcacgtggcgcttcactccggg ||||||||||||||||||..|.| gtcacgtggcgcttcactgggcg

Promoter sequences (retrieved from UCSC [30]), along with PEDB [32] and EMBOSS [33] were used to investigate CLOCK and BMAL1 transcriptional elements in the genes of interest. V# = Transcript variant number.

**Figure 2:**
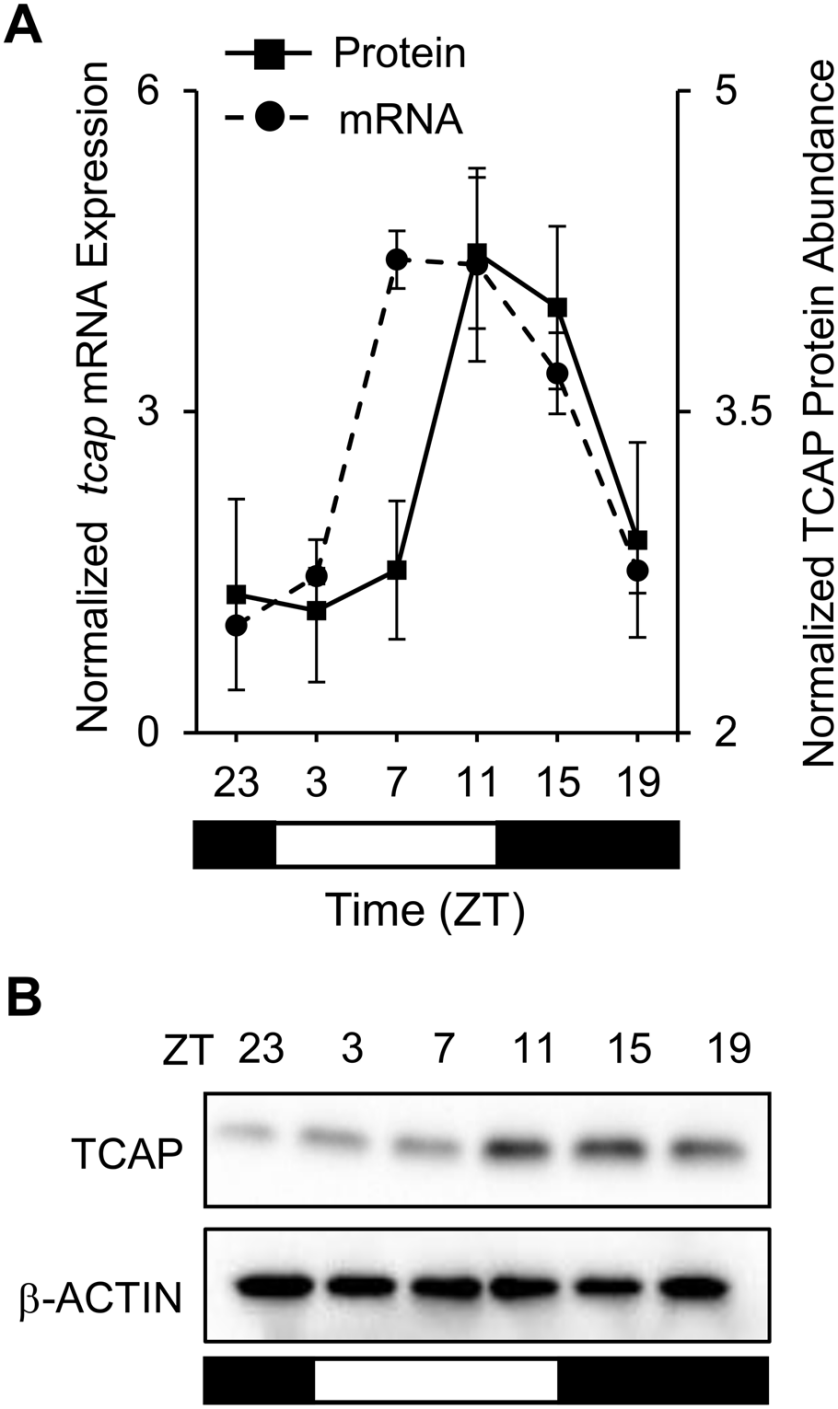
Diurnal cardiac *Tcap* mRNA and TCAP protein rhythms. Hearts were collected every 4 h across the 12∶12 L:D cycle from C57Bl/6N mice, and used for qRTPCR (mRNA) or Western blot (protein) analysis. (A) *Tcap* mRNA (dotted line) exhibited rhythmic expression (JTK_Cycle, p = 7.41×10^−5^) with a peak in the light phase at ZT07 (murine sleep time) and trough in the dark phase (n = 3/time point). TCAP protein (solid line) also exhibited a rhythmic profile (JTK_Cycle, p = 0.00139) that peaked in the light and reached a nadir in the dark (n = 3/time point). There was a 4 h phase delay between mRNA expression and protein abundance. (B) Representative Western Blot, illustrating TCAP protein abundance over the 12∶12 LD cycle. The diurnal environment of 12 h dark (black bars, animal’s subjective wake time) and 12 h light (white bars, animal’s subjective sleep time) is illustrated by the bars below the graphs.

In light of these findings, we experimentally validated our approach by investigating whether the *Tcap* gene, identified in all our lists, was a direct target of the circadian transcriptional activators CLOCK and BMAL1. Identification of *Tcap* as a circadian regulated gene was of considerable interest, as TCAP is a key component of the cardiac sarcomere and plays an important role in cardiac structure and function [15]–[17]. To demonstrate that *Tcap* mRNA expression exhibited a 24 h daily rhythm we first examined whether the mRNA was rhythmically expressed in murine heart under normal 24 h diurnal (12∶12 L:D) conditions. As shown in Figure 2A, *Tcap* mRNA exhibited a significant (p = 7.41×10^−5^) rhythmic profile by JTK_Cycle, that peaked during the light phase (murine sleep time) and reached a nadir in the dark (wake time). The TCAP protein profile was also rhythmic (p = 1.39×10^−3^) by JTK_Cycle across the diurnal cycle, and was visualized by Western blot analyses (Figure 2A, 2B). There was an ∼4 h phase delay between mRNA expression and protein abundance, consistent with the phase delay anticipated for core clock mechanism regulated proteins [40]. These data further support the notion that *Tcap* is a circadian regulated gene.

**Figure 3:**
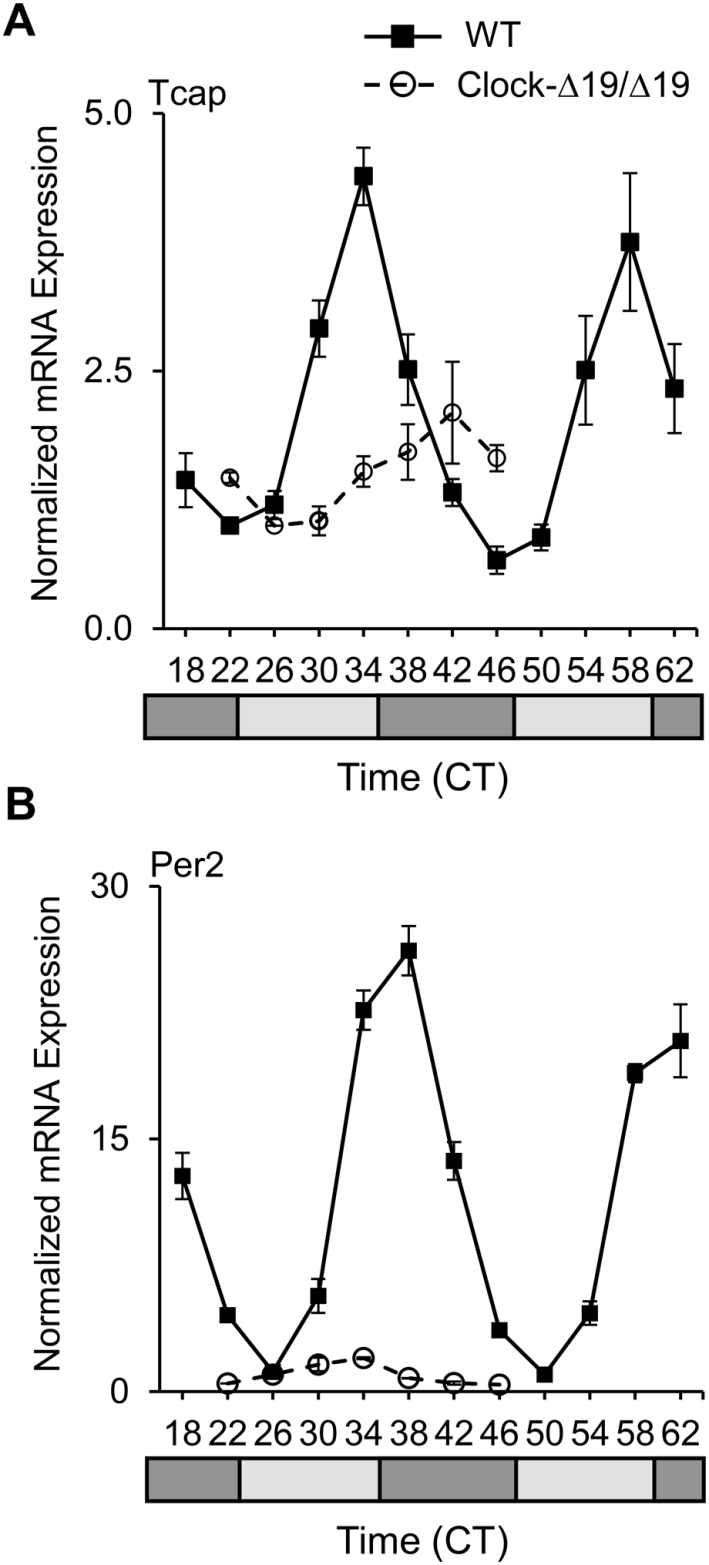
Circadian rhythms in *Tcap* and *Per2* mRNA expression in WT hearts, and blunted expression levels and amplitude in Clock^Δ19/Δ19^ heart. Starting at 30 h after transfer into D:D (CT = 18) the mice were euthanized, and hearts collected every 4 h from the Clock^Δ19/Δ19^ (○) and WT (▪) littermate mice (n = 4/time point). Rhythmic expression of (**A**) *Tcap* mRNA (JTK_Cycle, p = 1.09×10^−7^), and (**B**) *Per2* mRNA (positive control; JTK_Cycle, p = 1.18×10^−9^) in WT hearts. In contrast in Clock^Δ19/Δ19^ hearts, gene expression is barely periodic and amplitude is severely blunted consistent with being target genes. The circadian environment of 12 h dark (black bars, animal’s subjective wake time) and 12 h dark (grey bars, animal’s subjective sleep time) is illustrated by the bars below the graphs.

Since rhythmic diurnal oscillations can be due to circadian regulation or correlated to light influencing the major neuroendocrine systems (for example, [41]), and to distinguish and provide further support for circadian regulation, we next measured endogenous mRNA profiles under the circadian condition of constant darkness. As expected, *Tcap* mRNA exhibited a robust circadian rhythm (p = 1.09×10^−7^) by JTK_Cycle, in the hearts of wild-type (WT) littermate mice. The peak late in the subjective sleep time and trough late in the wake time (Figure 3A) correlated with the profile for *Tcap* from the microarrays (Mouse 1.OST Heart) on CircaDB. We then investigated a role for the circadian transcriptional regulatory factor CLOCK in *Tcap* mRNA expression, by examining the hearts of homozygous CLOCK-mutant (Clock^Δ19/Δ19^) mice. In contrast to WTs, Clock^Δ19/Δ19^ hearts exhibited an mRNA rhythm in *Tcap* that, while still apparent (JTK_Cycle, p = 0.02), was phase shifted and expression levels and amplitude were severely blunted consistent with it being a target gene (Figure 3A, Figure S1). Finally, we used the circadian mechanism CLOCK and BMAL1 regulated *Per2* gene as a positive control. As anticipated, *Per2* exhibited high amplitude endogenous cardiac mRNA cycling by JTK_Cycle (p = 1.18×10^−9^), and was similar to the microarray rhythmic profiles on CircaDB. Moreover, and as with *Tcap*, Clock^Δ19/Δ19^ hearts exhibited a weaker mRNA rhythm in *Per2* along with a severely blunted amplitude (Figure 3B), consistent with reports by others of blunted *Per2* rhythms in Clock^Δ19/Δ19^ SCN [23], [42]. Taken together, these findings are consistent with reports that mutant CLOCK protein associates with BMAL1 and binds to DNA but is deficient in transcriptional activity [23], [42], and further support the possibility that cardiac *Tcap* is a circadian regulated gene.

**Figure 4:**
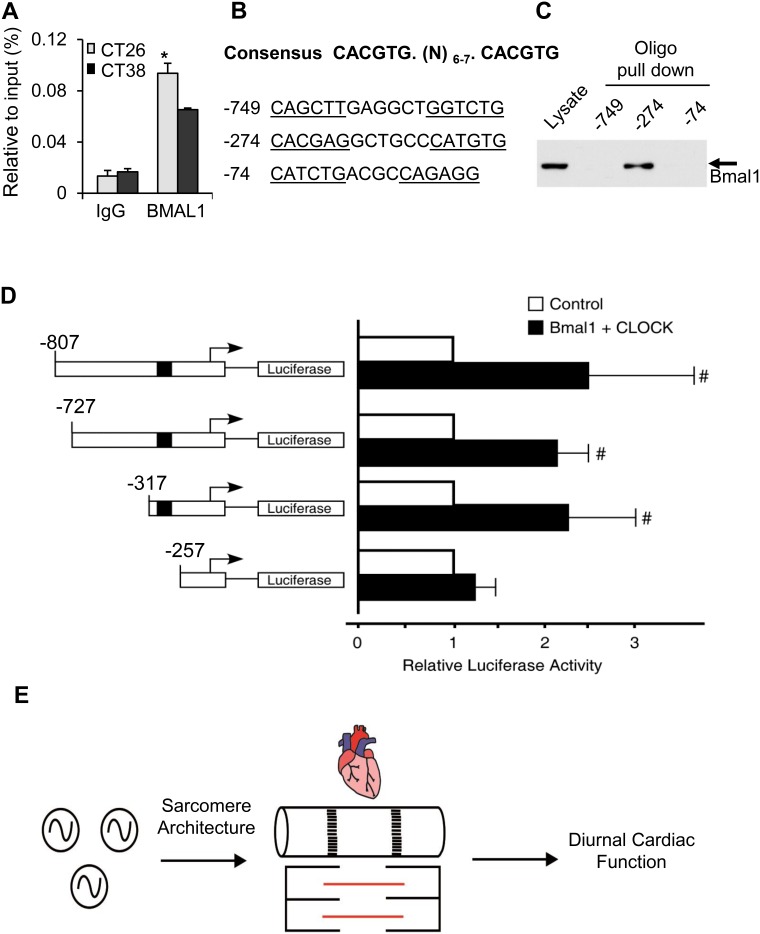
*Tcap* is a transcriptional target via binding to E-box motifs. (**A**) Chromatin immunoprecipitation (ChIP) assay performed using anti-BMAL1 antibody (n = 3/time point). Rabbit IgG was used as a negative immunoprecipitation control. (**B**) BMAL1 binding motifs in three E-box consensus sequences in the *Tcap* promoter, with location relative to the TSS noted. (**C**) BMAL1 was strongly precipitated by the −274 E-box, by DNA precipitation assays using biotinylated oligonucleotides. (**D**) BMAL1 increased *Tcap* promoter activity, whereas deletion of sequences to −257 (relative to the TSS, deleting the −274 motif, represented as the black box) abrogated increases in *Tcap* promoter activity, by luciferase reporter assay. Mean ± SEM, n = 4, # = p<0.05. (**E**) Illustration of circadian clock regulation of sarcomere architecture and thus diurnal cardiac structure and function. Left, (∼) = molecular clock mechanism; centre = cardiac sarcomere.

Finally, to test whether *Tcap* was a transcriptional target via binding to E-box motifs, we first performed chromatin immunoprecipitation (ChIP) assays using the E-box closest to the TSS (Figure 4A). The rationale for investigating this first site was that putative E-boxes can display a biased distance distribution from the TSS [32]. ChIP performed on heart samples collected when *Tcap* mRNA was increasing exhibited greater (p<0.05) binding (CT26, 0.094±0.008) as compared to when mRNA was decreasing (CT38, 0.065±0.001) (Figure 3B). In contrast, no binding was detected at the E-box using the control antibody, regardless of the time of day. However, given that binding was weak and exhibited only a 1.45 fold-change in amplitude over 24 h, and that ChIP fragments up to 500 bp may cover several putative binding sites, we therefore went for a different strategy and expanded our analyses to cover more of the upstream regulatory sequences in the *Tcap* promoter. To provide a more robust and definitive determination of *Tcap* circadian regulation we investigated three proximal E box sequences in the *Tcap* promoter located at −74 bp, −274 bp and −749 bp (Figure 4B). Notably, the putative E-box located at −274 bp matched very closely with the preferred BMAL1 binding motif, including the spacing and location immediately upstream of known BMAL1-regulated promoters [37]–[39]. To test whether the putative E-boxes bound to BMAL1, we performed DNA precipitation assays using biotinylated oligonucleotides. We found that BMAL1 was strongly precipitated by the E-box located at −274 bp upstream of the *Tcap* promoter. BMAL1 binding was not strongly detected in the DNA precipitates using the putative E boxes located at −749 or −74 bp upstream of the *Tcap* promoter (Figure 4C). These data indicated that the E box located at −274 bp upstream of the *Tcap* promoter recruits BMAL1. Next, we examined the function of E-box located at −274 bp upstream of the *Tcap* promoter using reporter gene assays. The expression of BMAL1 increased *Tcap* promoter activity (Figure 4D). Conversely, deletion of E-boxes to −257 bp (and thus the E-box located at −274 bp) abrogated increases in *Tcap* promoter activity (Figure 4D). Together, these data demonstrate that BMAL1 binds to at least the E box located at −274 bp, and regulates the *Tcap* gene promoter.

## Discussion

The cardiac transcriptome is comprised of hundreds of rhythmic genes that underlie the diurnal physiology of the cardiovascular system (for example, [11], [12]), however, which of these genes are directly regulated by the circadian clock mechanism in the heart is not known. In this study we generated an *in silico* approach to identify rhythmic heart-enriched genes likely to be under direct regulation by the positive loop of the circadian clock mechanism. The first step in the bioinformatics workflow was to identify genes with expression patterns similar to known clock controlled genes, by using the CircaDB website and JTK_Cycle algorithm. To focus on cardiac-specific processes, genes were then selected for high level cardiac expression, using the open access gene annotation portal BioGPS [28]. The promoter region of the resultant gene set was interrogated for conserved E-box motifs using UCSC, PEDB, and EMBOSS. We validated our approach towards identifying circadian regulated genes by experimentally investigating cardiac sarcomeric *Tcap* as a clock controlled gene.

One of the intriguing findings of this study was that cardiac *Tcap* transcription exhibited circadian regulation. TCAP (also known as titin-cap or telethon) is a 19 kDa protein component of the myocardial sarcomere [17], a cytoskeletal structure which is crucial for the mechanical and signaling functions of the heart. TCAP binds to the NH_2_-terminus of a giant and highly abundant protein termed TITIN (also called connectin, reviewed in [43]), at the Z-disc [44] which is the lateral boundary of the sarcomeric units in the cardiomyocyte cytoskeleton. TCAP is important for sarcomeric integrity and function [15], [16]. Since the heart exhibits diurnal variation in contractile performance [45], it is tempting to speculate that molecular circadian regulation of sarcomeric architecture contributes to cardiac function, as illustrated in Figure 4E. In support of this notion, genetic ablation of TCAP in mice altered t-tubules and led to contractile and stretch-sensing defects [46], and defects in TCAP Z disc complexes with muscle LIM protein were associated with dilated cardiomyopathy and heart failure in humans [47]. Further evidence for molecular circadian control of the sarcomere comes from studies that demonstrated that cAMP-dependent protein kinase (PKA) exhibited rhythmic mRNA expression [48], that diurnal disruption altered myofilament protein phosphorylation by PKA in a murine myocardial infarction model [49], and we and others recently demonstrated daily oscillations in cardiac myofilament function [31], calcineurin activity and protein phosphorylation [50], and in myocellular excitation-contraction coupling that relate to calcium homeostasis [51]. Further studies are clearly indicated to elucidate the precise mechanisms and clinical implications underlying circadian transcriptional regulation of sarcomeric structure and heart function. TCAP is also expressed in skeletal muscle [17], which may shed new light on the maintenance of muscle function in health and disease.

In this study, we used an *in-silico* bioinformatics approach along with *in vivo* and *in vitro* experimental validation to identify circadian regulated cardiac genes. Our analysis does not exclude the possibility that promoter ligands such as glucocorticoids [52] or NPAS2 [53] or other regulators [54], or diurnal variations in chromatin architecture (for example [55]–[59]) can also influence rhythmic cardiac gene expression. Indeed, this may help to explain the phase shift in gene expression in Clock^Δ19/Δ19^ hearts, which can be due in part to the difference in free-running circadian periods between WT (23.5 hr) and Clock^Δ19/Δ19^ (28 hr) mice in constant darkness (DD) [18], and could warrant future investigation. Genetic models have also been used to investigate circadian influences on cardiac gene expression, using cardiomyocyte-specific CLOCK mutant [48] and BMAL1 knockout [60] mice. However, regardless of the approach we observe the output of the clock mechanism as daily rhythms crucial to the cardiovascular system, such as the cyclic variation in heart rate, blood pressure, cardiac metabolism, and timing of onset of adverse cardiac events (reviewed in [1]–[4]). Understanding how the circadian mechanism regulates gene expression is important for providing a molecular and mechanistic basis for diurnal control of healthy cardiovascular function, and temporal control of pathophysiology in heart disease.

Our observations identified a wide variety of putative clock controlled genes, including *Tcap* which encodes a sarcomeric protein that plays an important role in cardiac structure and function. This approach is generally applicable to a wide range of tissues and organs, and can also be extended towards investigating the repressors, and other circadian mechanism complexes regulating circadian physiology in peripheral tissues.

## Supporting Information

Figure S1 **Log10 transformed data,** ***Per2*** **and** ***Tcap*** **mRNA, illustrating the magnitude of the Clock genotype effect.** The *Per2* mRNA profile (left) in Clock^Δ19/Δ19^ hearts is severely blunted in amplitude, consistent with *Per2* being a target gene. It is still periodic, but phase is advanced and expression levels and amplitude are greatly reduced. For *Tcap* in Clock^Δ19/Δ19^ hearts (right), again mRNA expression levels and amplitude are severely blunted, consistent with it being a target gene. Also, in Clock^Δ19/Δ19^ hearts, gene expression is barely periodic, which makes estimating phase and amplitude challenging.
(TIF)

Table S1 **94 rhythmic cardiac transcripts (p<0.001) with period 23–25 h and phase 16–20 h as determined by CircaDB.**
(XLSX)

Table S2 **Heart-enriched circadian genes selected using the BioGPS website, GeneAtlas MOE430 arrays.**
(DOCX)
