# Supplementary figures and images for: Circadian Regulation of Myocardial Sarcomeric Titin-cap (Tcap, Telethonin): Identification of Cardiac Clock-Controlled Genes Using Open Access Bioinformatics Data
Source: PLoS One. 2014 Aug 14;9(8):e104907. doi: 10.1371/journal.pone.0104907 (PMC4133362; doi:10.1371/journal.pone.0104907)

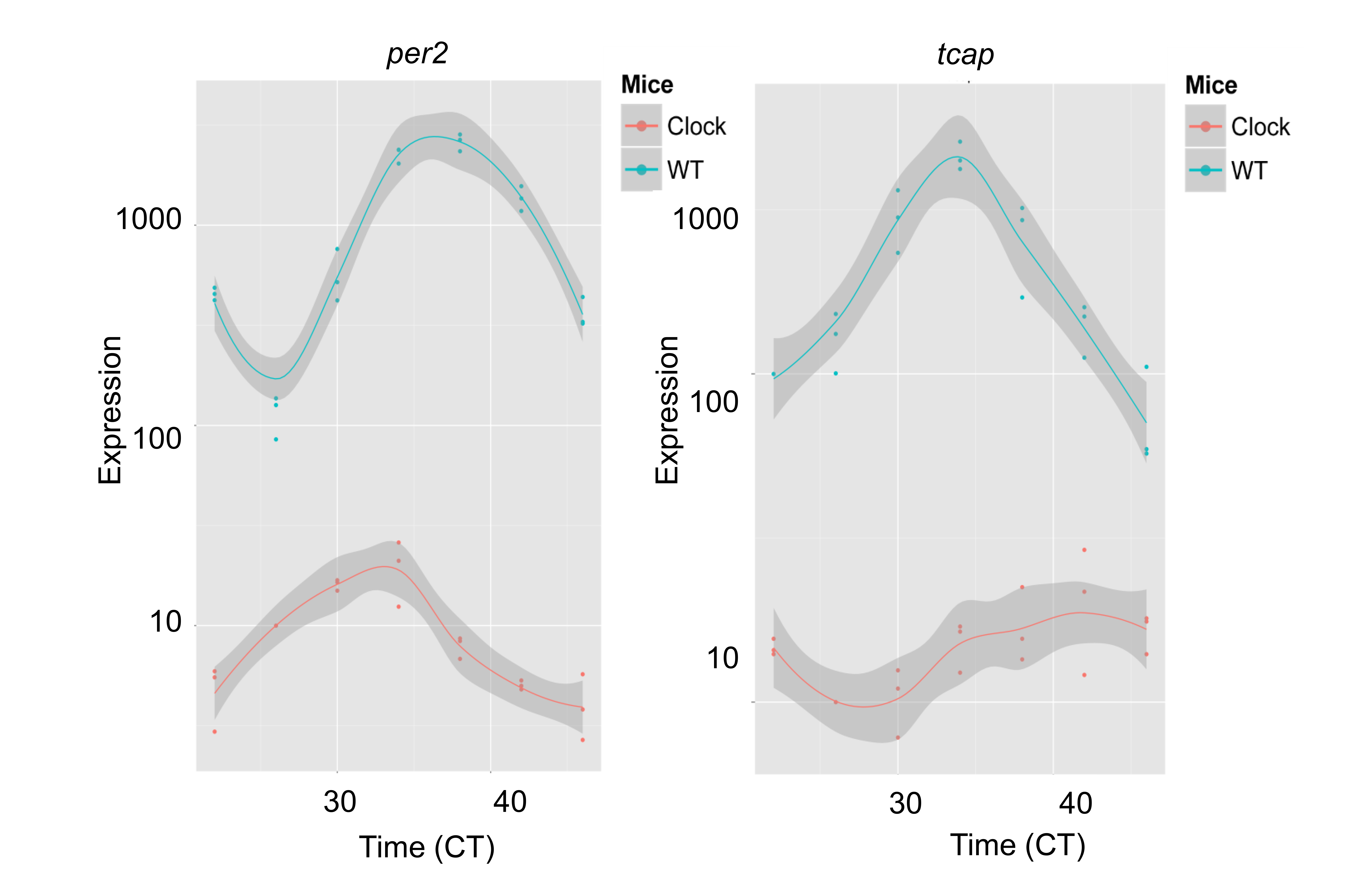

Supplement: Figure S1 — Log10 transformed data, Per2 and Tcap mRNA, illustrating the magnitude of the Clock genotype effect. The Per2 mRNA profile (left) in ClockΔ19/Δ19 hearts is severely blunted in amplitude, consistent with Per2 being a target gene. It is still periodic, but phase is advanced and expression levels and amplitude are greatly reduced. For Tcap in ClockΔ19/Δ19 hearts (right), again mRNA expression levels and amplitude are severely blunted, consistent with it being a target gene. Also, in ClockΔ19/Δ19 hearts, gene expression is barely periodic, which makes estimating phase and amplitude challenging. (TIF) [file pone.0104907.s001.tif]
