# Supplementary material for: Circadian Regulation of Myocardial Sarcomeric Titin-cap (Tcap, Telethonin): Identification of Cardiac Clock-Controlled Genes Using Open Access Bioinformatics Data
Source: PLoS One. 2014 Aug 14;9(8):e104907. doi: 10.1371/journal.pone.0104907 (PMC4133362; doi:10.1371/journal.pone.0104907)
Supplement: Table S2 — Heart-enriched circadian genes selected using the BioGPS website, GeneAtlas MOE430 arrays. (DOCX) [file pone.0104907.s003.docx]

**Table S2. Heart-enriched circadian genes selected using the BioGPS website, GeneAtlas MOE430 arrays.**

| **Gene** | **Probe ID** | **Cardiac expression levels** | **Average expression in murine tissues** |
| --- | --- | --- | --- |
| *Rhobtb1* | 1429206_at | 1940.8 ± 132.09 | 151.2 ± 19.6 |
| *Tcap* | 1423145_a_at | 5357.5 ± 2663.55 | 529.6 ± 179.2 |
| *Mlf1* | 1418589_a_at | 2869.8 ± 103.66 | 312 ± 102.9 |
| *Mylk4* | 1441111_at | 1853.9 ± 282.23 | 246.1 ± 118.9 |
| *Ccdc141* | 1429946_at | 95.9 ± 8.19 | 15.2 ± 1.2 |
| *Kcnh2* | 1449544_a_at | 177.6 ± 26.2 | 28.9 ± 4.6 |
| *Sh3rf2* | 1456640_at | 104.6 ± 29.4 | 20.3 ± 4.2 |
| *Gpcpd1* | 1429144_at | 4612.7 ± 1240.2 | 1023.3 ± 106.3 |
| *Calcoco1* | 1428513_at | 202.8 ± 31.22 | 48.1 ± 3.5 |
| *Ppip5k2* | 1433856_at | 1185.0 ± 350.4 | 298.9 ± 15.4 |
| *Vps25* | 1421050_at | 71.0 ± 35.1 | 18.1 ± 0.9 |
| *Raph1* | 1434302_at | 2590.3 ± 694.95 | 665.3 ± 57.1 |
| *Nfia* | 14211663_a_at | 596.34 ± 5.84 | 163.8 ± 20.6 |
| *Socs2* | 1418507_s_at | 1539.1 ± 627.6 | 443.3 ± 55.1 |
| *Cygb* | 1423630_at | 264.5 ± 32.0 | 80.7 ± 9.9 |
| *Klf9* | 1422264_s_at | 938.1 ± 19.22 | 296.9 ± 29.3 |
| *Usp2* | 1417169_at | 704.3 ± 164.5 | 272.5 ± 42.6 |
| *Dusp7* | 1452097_a_at | 608.01 ± 132.5 | 253.1 ± 17.2 |
| *1810013L24Rik* | 1434613_at | 2246.8 ± 145.0 | 972.9 ± 40.1 |
| *Acer2* | 1451355_at | 133.5 ± 3.52 | 68.8 ± 24.7 |
| *Slco5a1* | 1440874_at | 42.4 ± 11.48 | 23.3 ± 4.0 |
| *Timp3* | 1419088_at | 331.3 ± 145.03 | 203.5 ± 44.9 |
